# Methylene blue and malachite green dyes adsorption onto *russula delica*/bentonite/tripolyphosphate

**DOI:** 10.1016/j.heliyon.2024.e41250

**Published:** 2024-12-15

**Authors:** Ayfer Yildirim, Hilal Acay

**Affiliations:** a Vocational School of Health Services, Mardin Artuklu University, Mardin, Turkey; b Department of Nutrition and Dietetics, Faculty of Health Science, Mardin Artuklu University, Mardin, Turkey

**Keywords:** *Russula delica,* bentonite, Clay, Adsorption, Antimicrobial activity

## Abstract

In the current research *Russula delica* mushroom/bentonite clay (RDBNC) as a low-cost bionanosorbent was investigated for adsorption of methylene blue (MB) and malachite green (MG) dye from contaminated water. The bionanosorbent was characterized by Fourier transform infrared (FTIR), X-ray diffraction (XRD), Scanning electron microscopy (FESEM), Thermal Gravimetric Analysis (TGA), and Zeta-potential techniques. Adsorption experiments of RDBNC for MB, MG dyes following Freundlich isotherm and pseudo second order kinetic models. To determine their effects on the adsorption efficiency, the adsorption parameters were investigated including dye concentration, contact time, temperature, and dosage of the bionanosorbent. The adsorption process can operate through three primary mechanisms: the π–π interaction, the hydrogen bonding, and electrostatic interactions between the surface of RDBNC and MB, MG dyes. Desorption results revealed that MB and MG dyes were effectively desorbed during the fourth cycle without a notable loss in adsorption capacity. The thermodynamics parameters including ΔH, ΔS, and ΔG, were determined, and the adsorption process was favorable, spontaneous, and exothermic for MB and MG. The results showed that RDBNC, which showed effective inhibition at low concentrations, especially against *E. coli,* can be used as a low-cost bionanosorbent synthesised for the first time to remove industrial dyes.

## Highlights

• *Russula delica, mushroom/bentonite clay,* was synthesised for the adsorption of MB and MG.
• Kinetics and isotherm investigations were conducted for adsorption mechanisms.
• Gibb's free energy, enthalpy, and entropy were determined.
• The regeneration cycles were explored to evaluate the reusability of bionanosorbent.

## 1 Introduction

In recent years, synthetic dyes have been widely used in various industries, such as plastic, paper, food, medicine, and cosmetics, especially in the textile industries, due to their chemical stability and the ease and diversity of the synthesis process [1]. Unfortunately, these synthetic dyes are a potential threat that creates a severe toxic effect on the environment, and human and animal health (causing discomfort such as shortness of breath, rapid heartbeat, nausea, mental confusion, and skin irritation) when released into nature since they are generally non-biodegradable and bioaccumulative products even at low concentrations [2,3]. According to their ionic charge, synthetic dyes are divided into three groups: anionic, cationic, and non-ionic. MB and MG dyes are also included in the cationic dye group. In addition to causing gastritis, diarrhoea and skin diseases, MB used in the textile, cotton, silk, paper, fleece, and leather industries can cause damage such as cancer and genetic mutations in humans and other organisms [4]. MG is used for colouring wool, silk, and leather and also fungicide and treatment of protozoal infections and biocide in the aquaculture industry. Exposure to MG risks adverse events such as respiratory toxicity, mutagenesis, chromosomal breaks, and carcinogenesis of a non-biodegradable nature [[5], [6], [7]].

Due to the toxicity of dyes, an easy and cost-effective sustainable approach is urgently needed. The most commonly used methods for the removal of hazardous wastes from polluted water discharged from industries and domestic sewage are Flocculation [8], cation exchange membranes [9], ultrafiltration [10], adsorption [11], electrochemical degradation [12], oxidation [13]. In recent years, adsorption technology has been used to treat dye wastewater efficiently due to its simple operation and application, low energy and concentration applicability [14]. Biosorption, a cost-effective method, reflects the ability to remove dye from solutions using environmentally friendly and readily available adsorbents from agricultural waste, the food industry, and forestry [15].

*Russula delica* (RD) is a well-known macrofungi that grows in wooded habitats under both coniferous and deciduous trees. RD is taken every year when the weather conditions are suitable [16]. Recently, mushroom and mushroom waste have found their application as a biosorbent for dye wastewater treatment. For example, in the study of Sivasakthivelan [17], *Coriolus versicolor* was used as a biosorbent for acid green, dispersed red and basic orange dyes with 98, 76 and 61 % removal capabilities [17]. In another study, mushroom waste-derived g-C_3_N_4_ was used for MB adsorption, and the results showed that mushroom waste content improved the adsorption efficiency of MB [18]. Obtaining modified surfaces based on natural materials such as clay minerals, which are the most abundant ones on Earth associated with biomaterials such as RD, is a promising alternative to developing new and improved biosorbents. Clays provide many usage areas with their versatile properties such as porosity, large surface area, a laminated structure, specific active sites, high cation exchange capacity and also thermal stability. In addition, it is a very suitable material for modification to improve its adsorption capacities in providing improved materials for environmental pollution removal studies, thanks to its properties such as the exchange of cations in clay, heat treatments or acid treatments [19]. Bentonite, known as montmorillonite, is a naturally occurring clay variety that predominantly contains a smectite phase. In the literature, this clay is widely used in the synthesis of composites [20]. Ain et al. have synthesised a Fe_3_O_4_-Polydopamine composite with bentonite and investigated the adsorption performance of crystal violet, Rhodamine B, and Brilliant blue dyes.

When the literature is examined, not much innovation has been reported in the use of RD mushrooms in dye adsorption. In this context, the bionanocomposite prepared by supporting RD mushroom with clay will bring innovation to the literature content in the research on additive removal. Therefore, in the present work, a novel kind of bionanocomposite (RDBNC) has been synthesised using RD mushroom and bentonite clay with TTP for nanoscale and used for investigation of cationic dyes adsorption. Various adsorption experiments, the effect of time, temperature, pH, dye concentration, bionanosorbent dosage concentration, and regeneration were performed to evaluate the efficiency of RDBNC for MB MG removal. The isotherm, kinetic and thermodynamic parameters were also evaluated to understand the adsorption performance of the synthesised RDBNC bionanosorbent.

## 2 Materials and methods

### 2.1 Materials

Sodium tripolyphosphate (TTP) was purchased from Acros Organics (New Jersey, USA). Dimethyl Sulfoxide (DMSO) was supplied from Sigma-Aldrich. The acids and bases [sodium hydroxide (NaOH), Acetic acid (CH_3_COOH) and hydrochloric acid (HCl)] used in the experiments were at the analytical level. *Russula delica* mushroom samples collected from Mardin, Turkey, were dried in open air for about 15–20 days and then ground.

### 2.2 Preparation of RDBNC

4 g of RD and BNT were dissolved in 40 mL 1 % acetic acid and DMSO for 60 min, respectively (with an equal ratio). Then, both mixtures were added to each other, and the pH of the mixture was adjusted to 5.5 by HCl/NaOH (1 %) solutions. Separately, 0.8 g TTP was dissolved in 20 mL distilled water and poured into the RD/BNT mixture drop by drop. Subsequently, the RD/B/TTP solution was stirred with continuous and regular stirring for 2 h to obtain the nanoparticles. After 20–30 mL methanol was added to the mixture, the precipitation has occurred. Finally, the residue was filtrated with filter paper, dried overnight at 60 °C in the oven, and then sieved and labelled as RDBNC.

### 2.3 Characterisation of RDBNC

By using a Fourier Transform Infrared (FTIR) spectrophotometer using ALPHA Bruker (ZnSe crystal, Platinum-ATR accessory), the surface functional groups were investigated. A Rigaku Ultima-IV recorded the X-ray diffraction (XRD) pattern of RDBNC. The morphological properties of the RDBNC were inspected using a scanning electron microscope (FESEM, QUANTA 400F). SDT650 model Thermal Gravimetric Analysis (TGA) (30–950 °C), °C/min, N_2_ atmosphere, 100 ml/min) was used to demonstrate the thermal characterisation of RDBNC The zeta-potential technique was used to determine the particle size and zeta-potential analysis by the Malvern Nano ZS90 model Zeta potential device.

### 2.4 Batch adsorption studies

To explore the adsorption characteristics of RDBNC bionanosorbent concerning MB and MG dye adsorption, batch experiments were carried out. Batch adsorption was conducted by adding RDBNC to 100ml MB, MG solutions at room temperature, shaking at 140 rpm by a shaker water bath. By the batch of the experiments, the effects of contact time (0,30,45,60,90,120,180,240 min), initial MB concentration (25,50,100 mg/L), and adsorbent dose (10,20,30,40,50 mg) on the adsorption capacity of RDBNC bionanosorbent were investigated. To determine the optimum adsorption parameters, the adsorption kinetics of RDBNC bionanosorbent was investigated by using 10 mg bionanosorbent with three different initial MB, MG concentrations 25, 50, 100 mg/L and temperatures 30, 40, 50 °C. Also, isotherm studies were carried out with 10 mg of bionanosorbent with 25,50,75,100,125,150 mg/L initial MB, MG concentrations at ambient pH 6. All adsorption experiments were performed in 3 replicates and averaged. The dye concentration before and after adsorption was examined by a UV–Vis spectrophotometer (PG T80+ model) at 664 and 617nm for MB and MG, respectively. The % adsorption of dyes and the amount of dye adsorbed (qe) at equilibrium were calculated using eq. (1) and eq. (2).

(1) $$\%\;adsorption=\frac{Co-Ce}{Co}x100$$

(2) $$qe=\frac{\left(Co-Ct\right)xV}{m}$$

where Co, Ce and Ct are the dye concentration at initial, equilibrium and any t time (mg/L), respectively. *q*_*e*_ is the amount of dye adsorbed (mg/g), V is the solution volume (L) and m is the bionanosorbent mass (mg).

### 2.5 Antimicrobial efficacy studies

The inhibitory effects of synthesied RDBNCs on the growth of certain microorganisms were determined using the minimum inhibitory concentration (MIC) microdilution method according to Yıldırım et al. (2020) [21] for gram-negative (Escherichia coli ATCC25922), gram-positive (Staphylococcus aureus ATCC 29213) bacteria, and Candida albicans ATCC 10231 yeast. Muller Hinton medium, RDBNC solution, and a microorganism mixture prepared according to McFarland standard 0.5 (turbidity) were added to the microplate wells. The mixture was incubated at 37 °C for 24 h. To compare the antimicrobial effects of RDBNCs, standard antibiotics were used: vancomycin for S. aureus, colistin for E. coli, and fluconazole for C. albicans. Thus, the antimicrobial effects of RDBNC aqueous solution on microorganisms were investigated. The study was conducted in triplicate, and average values were determined as MIC values.

### 2.6 Characterisation of RDBNC

#### 2.6.1 FTIR

**Fig. 1:**
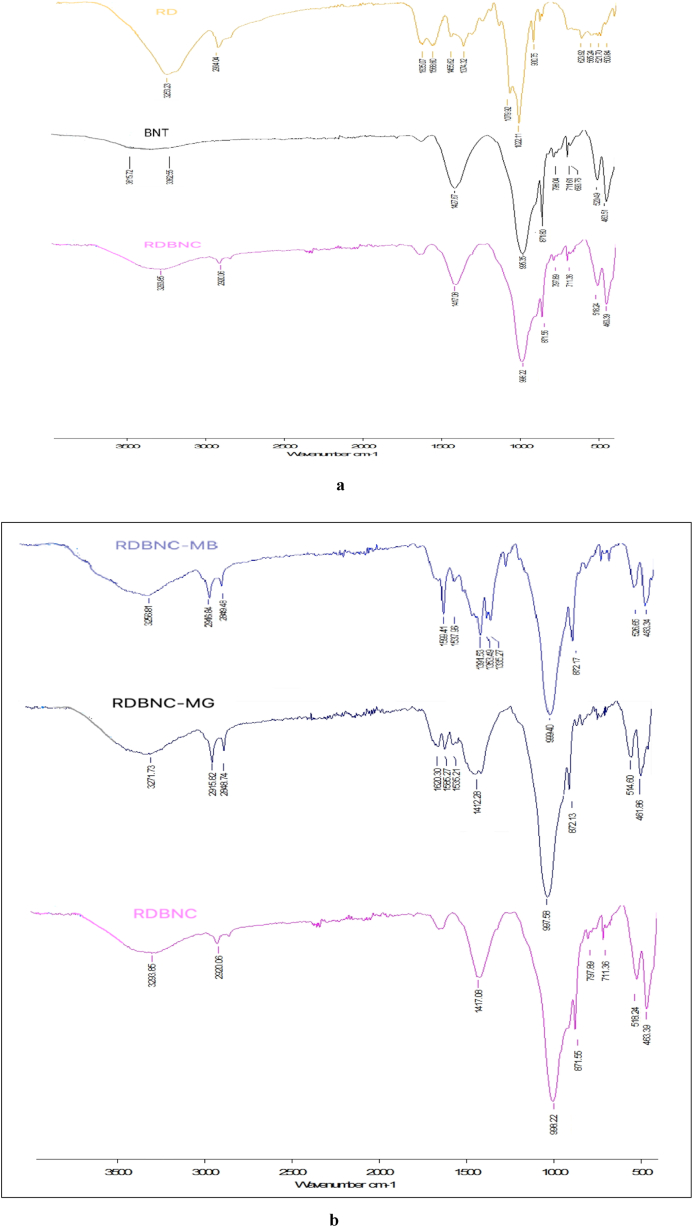
a: FTIR spectra of RD, B, and RDBNC **b:** FTIR spectra of RDBNC, RDBNC after MG and MB adsorption.

To identify the functional groups present in bionanosorbent, FTIR analysis is used. Fig. 1a depicts the resultant FTIR spectrum of RD, BNT and RDBNC. A broad band between 3500 and 3000 cm^−1^ is attributed to the -OH (Si-OH)/-NH stretching vibrations on the entire spectrum [[22], [23]].

In the RD spectrum, the bands correspond to typical -OH stretching vibrations and symmetrical and asymmetrical elongation of -CH_2_ groups are seen at 3253 cm^−1^ and 2923cm^−1^. The stretching vibration of the carbonyl group of mushrooms was responsible for the transmittance bands at 1635 cm^−1^ and 1566 cm^−1^ that indicated the presence of amide I (C=O) and amide II (C-N) (characteristic peaks of chitin and chitosan) reactive groups, respectively. A band at 1455 cm^−1^ is attributed to the aromatic stretching of the C=C group, while the bands identified at 1022cm^−1^ and 1079 cm^−1^ are related to C-C stretching vibration and the C-O asymmetric stretching vibration.

In the bentonite spectrum, calcite vibration is associated with the absorption band at 1427 cm^−1^, while quartz or Si-O-Si stretching vibrations of the clay mineral peak at 995 cm^−1^. Si-O is suggested to the band at 995 cm^−1^, whereas Al-OH-Al deformation vibration is assigned at 871 cm^−1^. Also, the band between 600 and 700 cm^−1^ can be assigned to the Al-OH deformation, while bands at 520 and 463 cm^−1^ show the characteristic elongation of the Si-O-Al bond (Fig. 1a).

In the bionanocomposite spectrum, the amide bands (1640 cm^−1^, 1598 cm^−1^) and aliphatic C-H group (2920 cm^−1^) region reflect the mushroom structure and the Al-OH, Si-OH vibration and elongation of Si-O-Al bands (1417 cm^−1^, 1644 cm^−1^ and 518 cm^−1^, 463 cm^−1^) reflect the clay structure. Shifting in these bands onto starting materials (RD, B) also supports this composite (RDBNC) formation. Thus, these similarities on the spectrum of RD, BNT and RDBNC support the reality of composite synthesis.

The spectra before and after loading MG and MB dyes onto bionanosorbent are depicted in Fig. 1b. As shown in Fig. 1b, renewed and shifting bands were observed in some band regions before and after the adsorption of the dyes onto bionanosorbent. The band that belongs to the O-H/N-H was shifted from 3293 cm^−1^ to 3271 cm^−1^ and 3281 cm^−1^ when the MG and MB dyes were adsorbed, indicating hydrogen bond between the OH/NH groups of RDBNC and the dye molecules [[24], [25], [26], [27]], suggesting that the OH/NH in the RDBNC bionanosorbent might have been the binding sites with MB and MG dyes.

After MG adsorption, while the peaks attributing the CH_2_ vibration groups became clear and showed shifts, at the same time, the amide bands became more prominent. Moreover, the formation of new bands at 1535 cm^−1^ and 1364 cm^−1^ belonging to the C=C aromatic ring and C-N aromatic tertiary amine group argues for the existence of adsorption of MG. After MB loading, the peaks exhibiting aliphatic C-Hs again became evident, the amide I band disappeared, and the amide II band became more distinct and shifted from 1598 cm^−1^ to 1599 cm^−1^. It was also determined that new peaks thought to belong to -CO-N and -CH_3_ were formed in 1537 cm^−1^ and 1391 cm^−1^, 1353 cm^−1^, and 1335 cm^−1^. Based on these changes in the bands of the spectra after adsorption (peak shifts, new peak formation), it can be emphasized that both cationic dye adsorption takes place on C-H (CH_2_), C-O, and RCONR_2_ groups.

#### 2.6.2 XRD

**Fig. 2:**
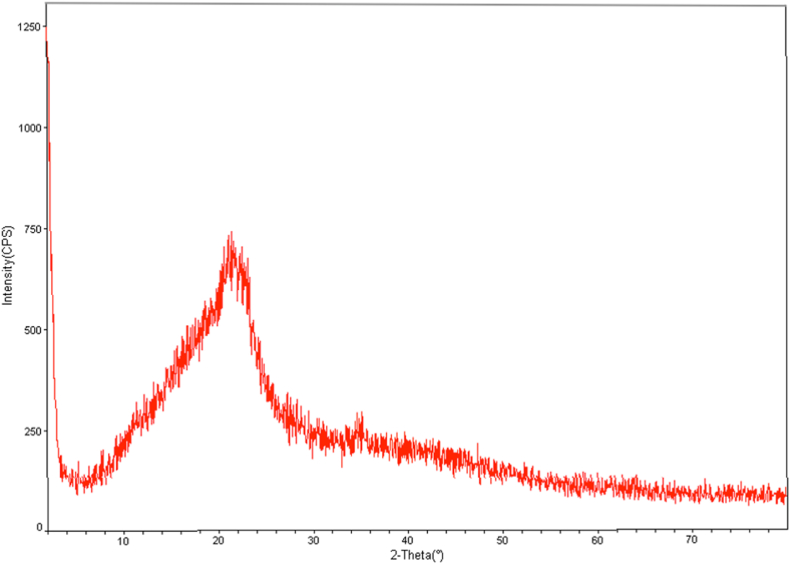
XRD pattern of RDBNC.

To determine the crystalline structure of RDBNC, XRD spectra were recorded. The XRD pattern of RDBNC is presented in Fig. 2. XRD analysis was done to determine the purity and phase growth of the bionanocomposite. According to Fig. 2, the XRD pattern of bionanocomposite showed a characteristic appearance of a small shoulder crystalline peak, indicating the presence of N-acetyl glucosamine sequences from chitin located at chitin/chitosan peak as 2θ = 21.5^o^ [28,29]. In addition, the distinct strong diffraction peak (2θ = 21.5^o^) indicates the presence of the RDBNC bionanocomposite's amorphous structure. Zhao et al. (2023) [28] reported that the XRD pattern of chitosan/Enoki mushroom foot polysaccharide composite cling film showed one broad peak at 2θ = 21^o^. The aggregate crystalline sizes of the samples were computed using an equation of Dybe Scherer. By using the D = (0.94 λ)/(β cos θ) equation (Dybe Scherer Eq.), the size of crystallite has been determined by the expansion of the diffraction line. In the equation, θ is equal to the peak position, wavelength represented by λ (λ = 1.542 Å) and β is equal to the FWHM of the line [30]. The crystallite size of RDBNC was calculated as 20 nm according to the Dybe Scherer Equation.

#### 2.6.3 TGA

**Fig. 3:**
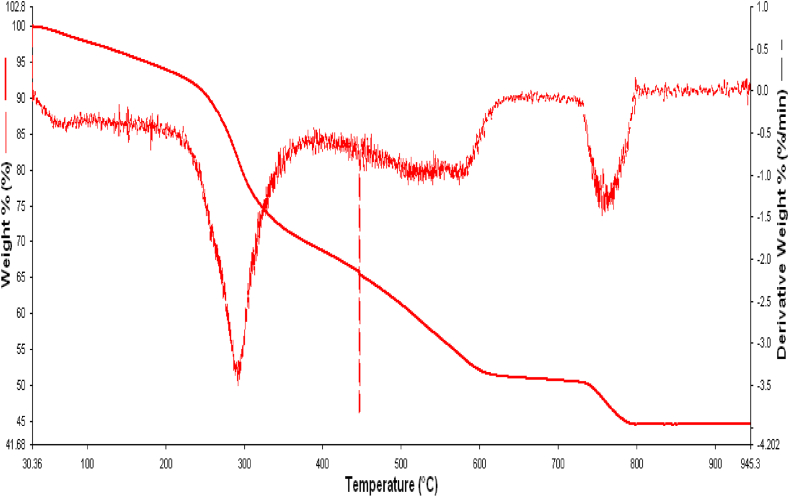
TGA analysis of RDBNC.

The thermal stability of RDBNC was examined by the TGA plot presented in Fig. 3. As a result of TGA analysis, the total mass loss of RDBNC was determined as 53.4 % in the range of 30–945 °C. Four degradation steps were discernible in the TGA curves of the RDBNC. The first stage represented the mass loose of tightly bound water evaporation and the degradation of low molecular weight compounds present in the mushroom's cell walls on the RDBNC at 30 to 100 °C. In addition, RDBNC is stable up to around 260 °C. The second and third stages of mass loss were between 260 and 310 °C and 310–600 °C. Weight loss at this stage was linked to processes that led to the degradation of the contribution of both β-glucans and chitin of the bionanocomposite that were conducted and completed together in agreement with that found for white button mushrooms (*Agaricus bisporus*) [31]. The degradation of clay is associated with the fourth stage between 750 and 800 °C. Wang et al. (2021) [32] reported the weight losses for clay mixtures that are demonstrated with dehydroxylation of kaolinite according to TGA results in the stage between 300 and 700 °C.

#### 2.6.4 FESEM

**Fig. 4:**
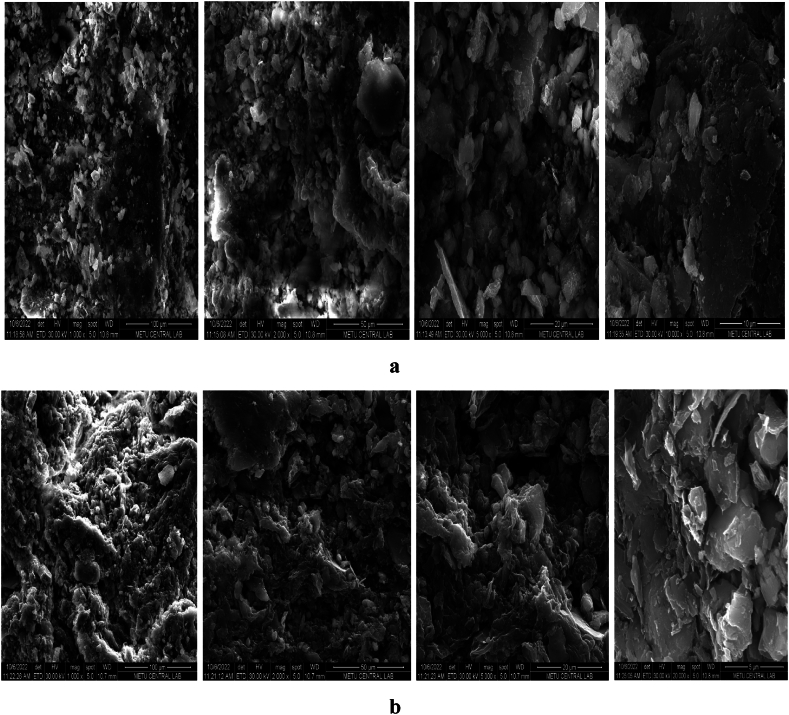

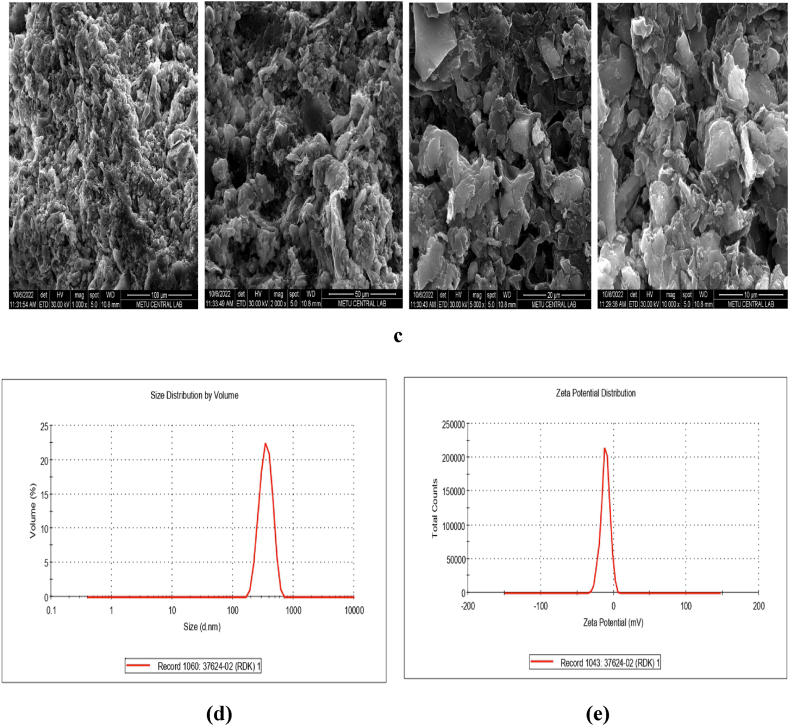
FESEM images of **a)** RDBNC before adsorption, **b)** RDBNC after MB adsorption **c)** RDBNC after MG adsorption, **(d)** Zeta-Potential Distribution and **(e)** Size Distribution by volume for RDBNC.

To determine the microstructure of particles of materials (morphology, texture, surface crystallinity and composition), FESEM can be used as characterisation. Fig. 4a–c exhibits SEM images of RDBNC before (a) and after MB (b) and MG (c) adsorption. According to Fig. 4a–c, the RDBNC surface consists of generally nonsmooth pores and uniform areas. In bionanosorbent images, the porous, rough, solid and permeable area of the clay surface also appears to be a blending of the irregular folds observed in the chitin/chitosan structure of the mushroom (Fig. 4a). In the images after both dye adsorption (Fig. 4b and c), it is seen that there is a significant change in the surface morphology. It is observed that both MG and MB have weak amorphous images before adsorption, and more clumped, more cavitated and sharp images are formed after adsorption. These observed changes are thought to be an indication that both dyes adhere to the RDBNC surface [33]. This indicates that the nanoparticle managed to effectively extract the target cationic dyes from aqueous solutions. Similar changes have been observed in the literature [34].

#### 2.6.5 Zeta-potential

Zeta-potential is a good index to consider particle interactions for dispersion stability in solution. Zero Zeta-potential charge implies no repulsive force between colloidal particles (at this time, colloidal agglomeration can occur), while the high value of Zeta-potential (positive/negative) implies a strong repulsive force. The Zeta-average particle size was 423.2 nm (Fig. 4d) while the Zeta potential value of RDBNC was −11.3 mV (Fig. 4e). The typical negatively charged surface of the clay and the OH groups of fungus-derived chitin/chitosan allow the surface of the bionanosorbent to be negative [35,36].

#### 2.6.6 The solution pH effect

**Fig. 5:**
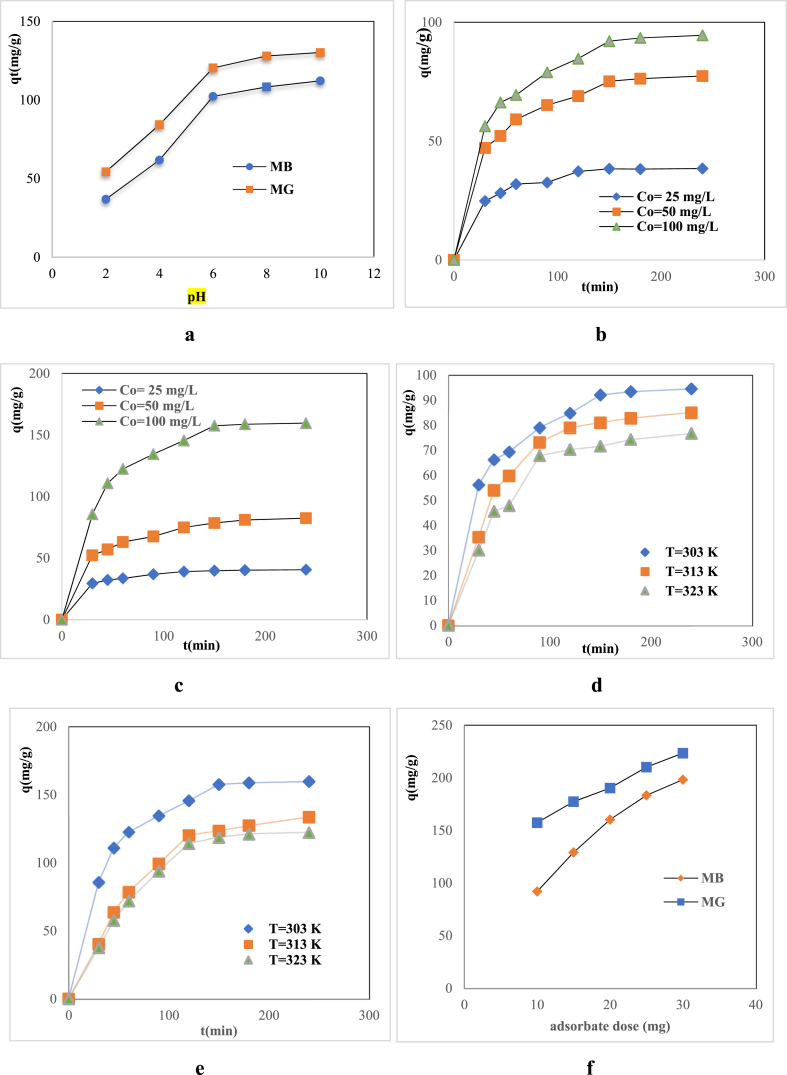
Effect of **(a)** pH **(b)** initial MB concentration **(c)** initial MG concentration **(d)** temperature (MB) **(e)** temperature (MG) onto RDBNC **(f)** bionanosorbent dose.

The effects of pH on MB and MG dye adsorption by RDBNC are shown in Fig. 5a. The solution pH effect can influence the protonation of biosorbent binding sites [37]. The fact that the zeta potential value is negative (−11.3 mV) indicates that the surface of RDBNC is negatively dominant. Thus, it can be said that the negative surface of the bionanosobent is quite suitable for cationic dye adsorption. As illustrated in Fig. 5a, the adsorption of MB and MG increased with the increase in pH. At low pH, electrically positive groups led to weak interaction between dyes and bionanosorbent formed by the amine group's protonation on RDBNC. As pH increased, the negative charge density of bionanosorbent was increased by protonation weakness, and so, the adsorption of both dyes increased. In the study conducted by Meskel et al. (2024) [38] using modified bagasse fly ash, they reached similar results in the pH effect on the adsorption of MG and MB dyes.

#### 2.6.7 The contact time, initial dye concentration and temperature effect

The adsorption of MB and MB dyes on RDBNC was examined as a function of contact time at an initial pH solution by using m = 0.01 g bionanosorbent, Co = 100 mg/L, V = 20 mL, T = 303 K and r = 140 rpm to determine the adsorption equilibrium time. The obtained results are depicted in Fig. 5b and c. According to the results, adsorption equilibrium was reached at approximately 150 min for both MG and MB dyes onto RDBNC. The RDBNC adsorption capacity was assessed with the initial MB, and MG concentrations at various contact times (0,30,45,60,90,120,150,180,240 min), as shown in Fig. 5b and c. According to Fig. 5b and c when initial MB and MG dye concentrations were increased from 25 to 100 mg/L, the adsorption capacity of both dyes onto RDBNC increased from 38.25 to 92.05 and from 39.76 to 157.53 mg/g for MB and MG, respectively. These increases in adsorption capacity with the initial dye concentration may be related to the greater concentration gradient in which, it serves as the driving force for the dye species to adsorb to the active RDBNC surface sites [39].

To examine the effect of temperature on RDBNC adsorption of dyes, three different temperatures of 303 K, 313 K and 323 K were investigated under the conditions of Co = 100 mg/L, m = 10 mg, V = 20 mL. Fig. 5d and e highlights the effect of temperature on adsorption capacity. Accordingly, it is conducted that the adsorption capacity decreases inversely as the temperature increases for MB, and MG dye adsorption onto RDBNC which is expected for an exothermic process. In MB adsorption, it was observed that the dye adsorption capacity decreased from 92.05 mg/g to 71.59 mg/g when the temperature increased from 303 K to 323 K. In the case of MG adsorption, adsorption capacity decreased from 157.53 mg/g to 118.52 mg/g with an increase in temperature from 303 K to 323 K. This shows that the increase in temperature is not suitable for the removal of dyes. Due to the exothermic reaction, high operating temperatures should be avoided to promote interactions between MB and MG dyes and RDBNC adsorption sites. Consequently, further experiments were conducted at a temperature effect [40]. Table 1 depicts the different adsorbents in the literature for MB and MG dyes. According to adsorbent capacities of adsorbent in Table 1, the RDBNC bionanosorbent is suitable for MB and MG dyes. Our findings highlight the potential of the obtained RDBNC bionanocomposite as both a new and good alternative to the limited use of fungus/clay composite and a sustainable and new material for the recovery and reuse of MG and MB dyes in wastewater treatment.

**Table 1:** Comparison of MB, MG adsorption onto different adsorbents in literature.

Adsorbent	Adsorbate	Adsorption capacity (mg/g)	Reference
smooth clay, rigorous clay, bentonite clay	MB	147.64	[41]
manganese dioxide decorated composites	MB	128.5	[42]
functional hydrogel	MB	222.65	[43]
magnetic core-double-shell structure	MB	34	[44]
chitosan-modified MMT	MB	158.79	[45]
hollow mesoporous hydroxyapatite	MB	143	[46]
RDBNC	MB	92.05	This study
surfactant-tailored alginate hydrogel beads:	MG	700	[47]
modified Bagasse Fly Ash	MG	15.5	[38]
pine gum hydrogel	MG	120.772	[48]
calcium silicate (CS) nanopowders	MG	13.44	[49]
jackfruit peel based activated carbon	MG	66.80	[50]
plant-based material	MG	24.8	[51]
RDBNC	MG	157.53	This study

#### 2.6.8 Bionanosorbent dose effect

The amount of bionanosorbent (dose) which is a critical factor in the adsorption process influences the adsorbent capacity. Fig. 5f illustrates the effects of the RDBNC dose on the adsorption capacity for MB and MG dyes. To assess the consequences of the bionanosorbent dose, RDBNC with doses ranging between 10 mg and 30 mg with Co = 100 mg/L, V = 20 mL dye solution, T = 303 K, and r = 140 rpm conditions were used. As illustrated in Fig. 5f, the dose of RDBNC rose from 10 mg to 30 mg, the bionanosorbent adsorption capacity increased from 92.05 mg/g to 198.33 mg/g and from 157.5 mg/g to 223.54 mg/g for MB and MG dyes, respectively. There is an increment in the adsorption of both MB and MG dyes with a rise in the amount of bionanosorbent. It could be explained by that the supernal amount of bionanosorbent provides more surface area to engage with dye molecules. Also, increasing in the bionanosorbent dose ensures the number of available active sites on the bionanosorbent [52].

#### 2.6.9 Adsorption kinetic

Two kinetic models were adopted, Langergen pseudo first order, and Ho and McKay pseudo second order kinetic models to illustrate the mechanism and rate of MB and MB adsorption onto RDBNC bionanosorbents [53]. To estimate the rate of MB and MG dye adsorption at the RDBNC bionanosorbent surface, both models were employed. The linear regression of the pseudo first order (3), and the pseudo second order (4) are given below.

(3) $$\ln\left(q_{e}-q_{t}\right)=\ln\;q_{e}-k_{1}t$$

(4) $$\frac{1}{q_{t}}=\frac{1}{k_{2}{q_{e}}^{2}}+\frac{t}{q_{e}}$$

q_e_ is the dye adsorbed (mg/g) at equilibrium, and q_t_ represents the adsorption at time t (min). *k*_*1*_ (1/min) is the rate constant for the pseudo first order kinetic model while *k*_*2*_ (g/mgmin) for the pseudo second order kinetic model

(5) $$k_{2}=k_{\exp}\;\left(-\frac{Ea}{RT}\right)$$

where *k* (g/mgmin) is the temperature-independent constant, and *Ea* (kJ/molK) is the adsorption activation energy.

**Fig. 6:**
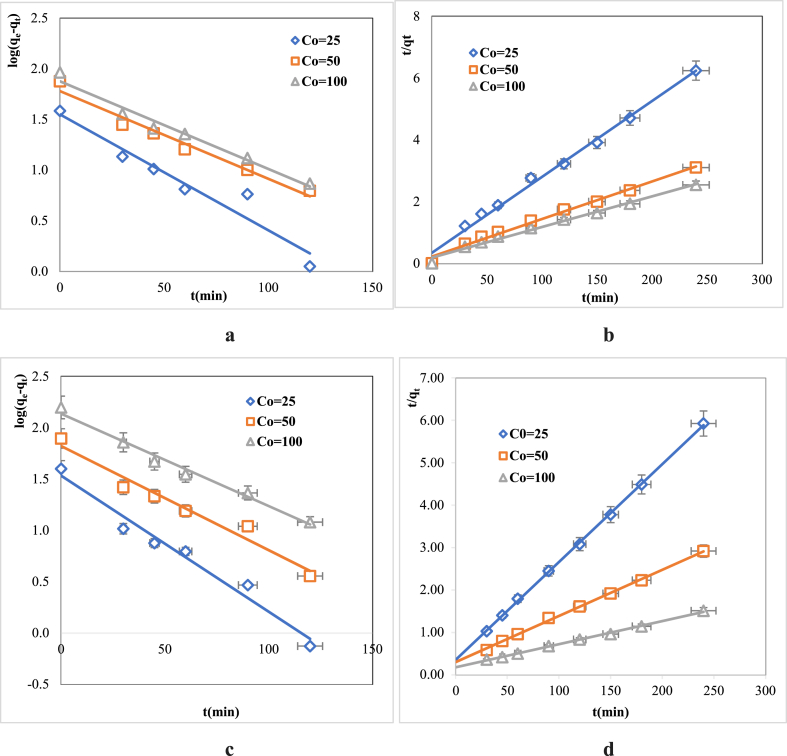
Pseudo first order (**a**:MB, **c**:MG), pseudo second order (**b**:MB, **d**:MG) kinetic models (C_o_ = 25–100 mg/L).

By drawing Pseudo-first and Pseudo-second-order curves fitted to the obtained experimental data, R^2^ the correlation coefficient and the corresponding parameters of the relevant rate laws are thus calculated. The pseudo first order and pseudo second order kinetic models can provide whether adsorption is physisorption or chemisorption. Both theoretical and experimental kinetic adsorption results of MB, and MG dyes onto RDBNC are depicted in Fig. 6a,b,c,d with the corresponding data presented in Table 2. As shown in the Tables, the experimental data points of adsorption of both MB and MG dyes on RDBNC were better compatible with the pseudo second order model than the first-order kinetic model. The higher R^2^ (0.98–0.99) values for the pseudo second order model and the counted (*q*_*e,c*_) adsorption capacities closely matching the experimental values (*q*_*e,exp*_) indicate a strong affinity for the pseudo second order model. Based on kinetic results, the MB and MG adsorption with RDBNC followed the pseudo second order model, proposing that these two dye adsorptions occurred through chemical adsorption indicating the adsorption process was the rate-limiting step and thus affected rates of adsorption-desorption [54]. Safdarian et al. (2024) [55] have been reported counterpart findings. The results showed that the data were best fitted by the pseudo second order model, with physisorption as the rate-limiting step.

**Table 2:** The pseudo first order, pseudo second order kinetics data.

Adsorbate			Pseudo first order			Pseudo second order		
	Co(mg/L)	*q*_*e,exp*_ (mg/g)	*q*_*e,c*_ (mg/g)	*k*_*1*_ (1/min)	R^2^	*q*_*e,c*_ (mg/g)	*k*_*2*_ (g/mgmin)	R^2^
**MB**	25	38.35	35.36	0.0263	0.9335	40.65	2.9533	0.9923
	50	75.14	60.03	0.0200	0.9700	81.97	4.5579	0.9891
	100	92.05	75.08	0.0200	0.9738	101.01	5.4348	0.9883
**MG**	25	39.75	34.03	0.0306	0.9718	43.29	2.8580	0.9996
	50	78.46	66.30	0.0235	0.9631	91.74	3.4235	0.9984
	100	157.53	136.87	0.0207	0.9841	171.82	5.9666	0.9976

**Fig. 7:**
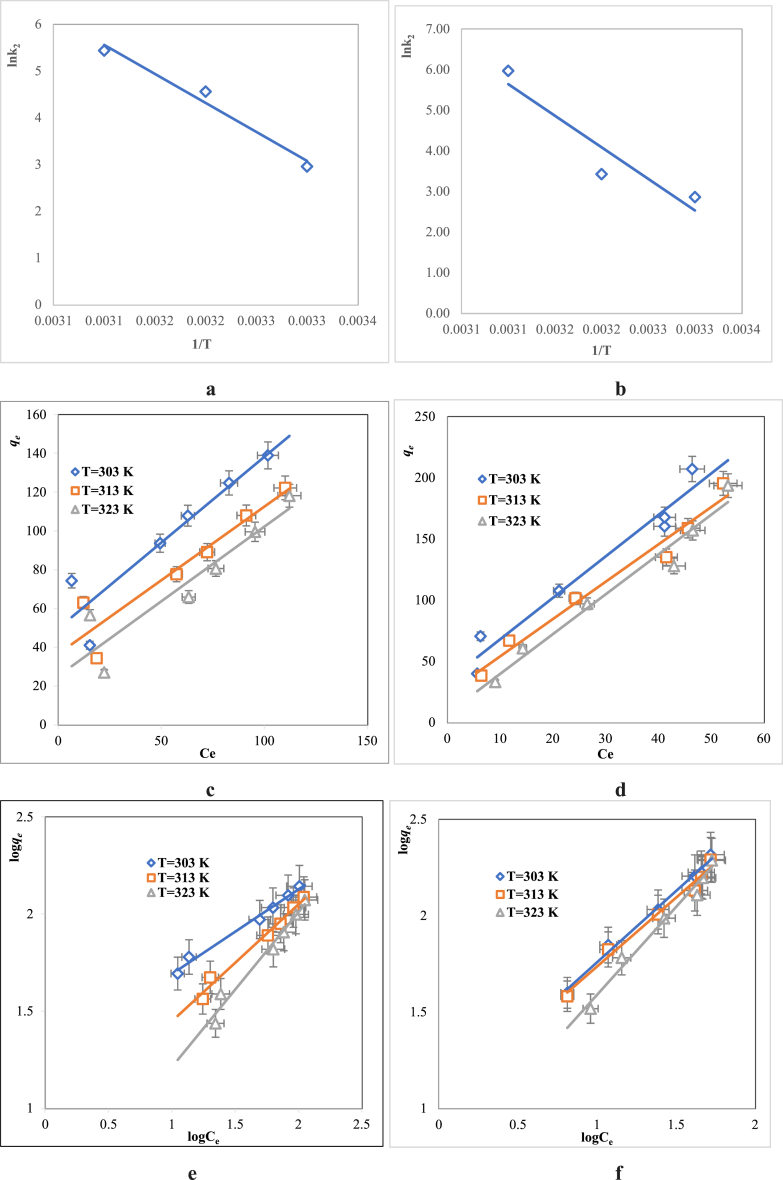
Plots for *Ea* calculation: **a**(MB)**, b**(MG)**,** Langmuir: **c**(MB), **e**(MB), and Freundlich: **d**, (MG), **f** (MG).

The value of *Ea* acquired from the Arrhenius equation can be used to identify whether the adsorption mechanism is physical or chemical adsorption. In physical adsorption, the activation energy is between 5 and 40 kJ mol^−1^ whereas the values exceed 40 kJ mol^−1^ in chemical adsorption indicating a more stable chemical bond. As presented in Fig. 7a, The activation energy, the adsorption rate constant (*k*_*2*_) of the pseudo second order model, and the adsorption temperature are the relevant factors that form the Arrhenius equation.

The calculated *Ea* was 103.1 kJ mol^−1^ for MB, and 129.2 kJ mol^−1^ for MG higher than the value of 40 kJ mol^−1^ indicating possibly stronger bond formation and thus chemical adsorption occurred at the MB, and MG dye adsorption onto RDBNC. A different study utilized Fe_2_O_3_@BC-KC composite to study the adsorption of Alizarin red S dye [56]. This conclusion was supported by the calculated *Ea* value of 65 kJ mol^−1^, which falls within the typical chemical adsorption value > 40 kJ mol^−1^.

#### 2.6.10 Adsorption isotherm

To describe the binding dye ions with the RDBNC bionanosorbent surface, adsorption isotherms analysis is a very important parameter. Two isotherm models, Langmuir and Freundlich, were considered to investigate isotherm [57].

The nonlinear equation of the Langmuir is presented in Equation (7).

(7) $$\frac{C_{e}}{q_{e}}=\frac{1}{q_{e}K_{L}}+\frac{C_{e}}{q_{m}}$$

While *K*_*L*_ is the Langmuir constant, *C*_*e*_ is the equilibrium concentration of the dye, and *q*_*m*_ is related to the adsorption capacity of the bionanosorbent.

The Freundlich isotherm is expressed by Equation (8).

(8) $$\log\;q_{e}=\log\;K_{F}+\frac{1}{n}\log\;C_{e}$$

Here *K*_*F*_ (L/mg) is related to the adsorption capacity and indicates the equilibrium constant, the 1/n is the heterogeneity factor and determines the bionanosorbent-dye interaction density surface.

As seen in Table 3, obtained from Fig. 7c,**d**,**e**,**f**, a high value of the correlation coefficient (R^2^) 0.97–0.98 for MB, and 0.97–0.99 for MG indicates good goodness of fit to the Freundlich isotherm model. A study showed nearly results that investigated the removal of crystal violet dye using zero-valent nickel/nickel oxide@graphene nanosheets [58]. Freundlich isotherm model describes the adsorption behaviour of dye molecules on the surface of bionanosorbent assuming occurring of multilayer-adsorption and emphasizing an exponential decrease in the energy distribution of the adsorbed sites and it also represents the intense binding between the MG, MB dye molecules with RDBNC bionanosorbent surface. Therefore, it confirms that multilayer adsorption of MB and MG dyes occurs on the surface of RDBNC bionanosorbent. A value of 1/n between 0 < 1/n < 1 indicates suitability for adsorption while close to 0 indicates that the surface may be more heterogeneous. In addition, the value n > 1 is related to physical adsorption while n < 1 is related to chemical adsorption. Therefore, when the adsorption results of both dyes are examined, it is emphasized that the adsorption mechanism of MB, and MG dyes on the RDBNC surface is physical adsorption and intense binding between dyes and bionanosorbent since n > 1, as seen in Table 3. In addition, the intercept and slope of the plot of In*qe* vs. ln*Ce* (in a straight line), give the values of *k*_*F*_ and 1/n respectively (Fig. 7e(MB), f(MG)). From the value of the slope of the graph in Fig. 7e and f (1/n) was found to be 0.436 (303K), 0.6 (313K), 0.7985 (323K) for MB and 0.74(303K), 0.71(313K), 0.912 (323K) for MG, respectively, in the range of 0 < 1/n < 1, and therefore both cationic dyes reflect proper adsorption by the RDBNC bionanosorbent. Also, the value of the *K*_*F*_ constant related to the binding energy was found higher at low temperatures (313 K) for both MB, and MG dyes, and this value indicates the higher affinity of dye molecules towards the RDBNC surface (Table 3).

**Table 3:** Constants of Langmuir and Freundlich isotherm.

Dye	T(K)	Langmuir			Freundlich		
		*q*_*m*_	*K*_*L*_	R^2^	*K*_*F*_	*n*	R^2^
MB	303	1.12	0.017	0.8638	17.98	2.294	0.9886
	313	1.12	0.035	0.8833	6.98	1.658	0.9834
	323	1.06	0.097	0.8590	2.59	1.252	0.9705
MG	303	0.29	0.100	0,9571	10.48	1.351	0.9915
	313	0.32	0.131	0.9714	10.40	1.398	0.9831
	323	0.30	0.467	0.9658	4.74	1.096	0.9788

#### 2.6.11 Thermodynamic parameters

(9) ΔG=−RTlnK

(10) $$\ln\;K=\frac{\Delta S}{R}-\frac{\Delta H}{T}\left(\frac{1}{T}\right)$$

Here, ΔG is Gibb's free energy, while ΔH and ΔS is the enthalpy and entropy of the adsorption mechanism, respectively.

**Fig. 8:**
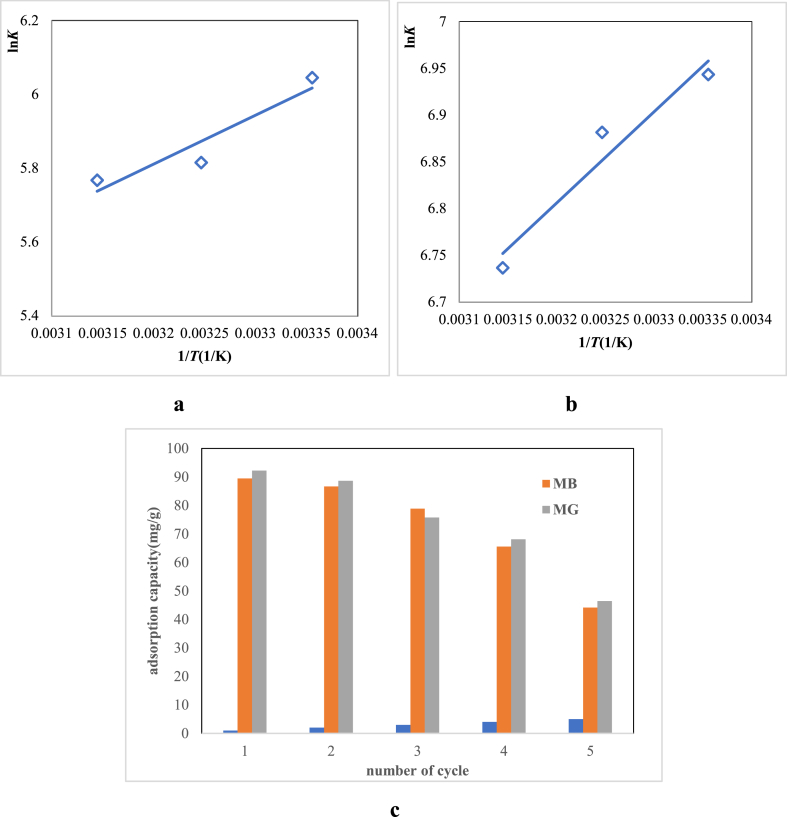
Van't Hoff plot for **a:**MB, **b:**MG; **c:**%reusability of RDBNC for MB, MG.

The ΔH and ΔS values can be estimated from the slope and intercept of the Van't Hoff plot, respectively [59]. Fig. 8a and b shows the Van't Hoff plot of ln*K* vs 1/*T*. All the thermodynamic parameters of the MB and MG adsorption on the RDBNC are given in Table 4. The negative ΔG values imply a favorable and spontaneous adsorption confirming that the adsorbent and the adsorbate will tend to interact and bind together. Moreover, the ΔH values of both dyes adsorption on the RDBNC are −11.02 kJ mol^−1^, and −8.2 kJ mol^−1^, respectively, indicating the physical adsorption (<80 kJ mol^−1^). The negative values of ΔH for both MB and MG illustrate the exothermic nature of the adsorption process. Also, the ΔS values of MB, and MG adsorption onto RDBNC are positive 1.57, 3.6 J/molK, respectively, suggesting that the adsorption mechanism of both dyes is an entropy-controlled process with greater randomness at the bionanosorbent/dye solution interfaces. The same result was inducted in the literature [60].

**Table 4:** Thermodynamic parameters.

Dye	T(K)	ΔG(−)(kJ/mol)	ΔH(−)(kJ/mol)	ΔS(J/molK)
MB	303	15.23	11.02	1.57
	313	15.13		
	323	15.49		
MG	303	17.49	8.2	3.6
	313	17.91		
	323	18.09		

#### 2.6.12 Mechanism

Three potential mechanisms, hydrogen bonding, π–π interactions, and electrostatic interactions can be interpreted for involved contact interactions between RDBNC and dye ions [61]. The adsorption process of dyes onto RDBNC involves significant functional groups: hydroxyl, carboxyl and amino groups. FTIR analysis of RDBNC before and after MB and MG adsorption was compared to understand the feasible adsorption mechanism. The stretching bands of the NH/OH group shifted from 3293 cm^−1^ to 3271 cm^−1^ and 3281 cm^−1^ after MG and MB adsorption, suggesting that the NH/OH groups in the RDBNC bionanosorbent might have been the binding sites with dyes. Absorption peaks near 1417 cm^−1^ were associated with Al-OH groups, and the absorption peak near 998 cm^−1^ represented the C-O (C-O-C) stretching vibration of the RDBNC were shifted to 1427 cm^−1^, 1455 cm^−1^, and 997 cm^−1^ and 999 cm^−1^ for MG, MB adsorption, respectively, confirming the effective adsorption of MG and MB onto RDBNC. In addition, these shiftings suggested that the C-O and Al-OH in the RDBNC bionanosorbent may have been the binding sites with both dyes. The characteristic peak of the amine/hydroxyl groups shifting promoted the hydrogen bonding interaction occurrence. After MG and MB adsorption, the characteristic peaks of the C=C (benzene ring) stretching vibration at 1590 cm^−1^ shifted to 1585 cm^−1^ and 1599 cm^−1^, presuming the involvement of the π bonds of aromatic delocalized of MG and MB with π-π interaction. This confirmed the adsorption between the RDBNC and dye molecules. Both MG and MB cationic dye are positively charged (MB^+^, MG^+^), and the RDBNC surface was negatively charged (OH^−^) according to Zeta-potential results (−11.3 mv). In the adsorption of MG and MB dyes on RDBNC, electrostatic repulsion-attraction played an important role. Hereby, the negative charged surface of RDBNC bionanosorbent exhibited strong electrostatic attraction with the positively charged organic dyes. Hydrogen bonding occurred between the O atom of the H acceptor and the OH/NH groups on the RDBNC surface.

Enthalpy values that are <80 kJ mol^−1^ involve the physical adsorption between RDBNC and MB, MG dyes.

The results also showed that the adsorption capacity for the MG was higher than that of the MB. A higher adsorption capacity indicates that a reaction is more likely to occur due to a more stable adsorption site for MG dye ions.

#### 2.6.13 The reusability of RDBNC

Reusability is considered an important factor in evaluating the utility of a bionanosorbent in practical applications. According to Fig. 8c, the recyclability study of RDBNC was conducted over four adsorption/desorption cycles. Desorption efficacy was evaluated through batch adsorption experiments using 0.1M HCl, and 0.1M NaOH. After each cycle, the sulfonated RDBNC bionanosorbent was completely separated from the solution using centrifugation, followed by soaking in HCl. The obtained results, as given in Fig. 8c, indicate that 89.5 % and 92.2 % of MB and MG, respectively, were effectively desorbed without a notable loss in adsorption capacity during the first cycle. However, in the fifth cycle, the desorption percentage decreased to 44.14 % (MB), and 46.47 % (MG) implying a gradual decrease with the cycle as the number increases (Fig. 8c). Despite this decrease, the RDBNC demonstrated successful reusability up to the fifth cycle, highlighting its potential for sustained application.

#### 2.6.14 Determination of the antimicrobial effect of RD and RDBNC

The development of resistance of pathogenic microorganisms against existing antibiotics increases the importance of the effectiveness of new-generation antimicrobial agents. The antimicrobial activity of the bionanocomposite synthesised in this study was demonstrated by using the minimum inhibition concentration (MIC). RD and RDBNC showed significant (Table 5). In the antimicrobial effect on microorganisms at lower concentrations compared to BNT and antibiotics study, RDBNCs (0.12 mg/mL) were found to be much more effective, especially on Escherichia coli. The sensitivity of the microorganisms used in the study to RDBNCscompared to antibiotics can be ranked as Escherichia coli (0.12) > Stapylococcus aureus (0.25) > *Candida albicans* (0.62). It was also found significant that RD showed very strong antimicrobial activity against *Candida albicans* (0.06) and Staphylococcus aureus (0.12) compared to antibiotic and B. This indicates that synthesised RDBNC and RD can be evaluated as an alternative microbial agent at lower concentrations. The study is in agreement with many studies in the literature [21,62]. In their research, Acay and Yıldırım (2022) [63] reported that the RDEK nanocomposite, synthesised from the extract of *Rusulla delica* (RD) and bentonite clay (K), exhibited considerable efficacy against the plant pathogen *Verticillium dahliae* (OVd82), demonstrating an inhibition zone of 21 mm. In this regard, the RDBNC nanocomposite, produced through comparable methodologies in the present study, underscores its versatility in antimicrobial activity.

**Table 5:** MIC values of Synthesied RDBNC, RD and BNT (mg mL^−1^) on vancomycin, fluconazole, colistin antibiotics, S. aureus, E. coli, and C. albicans microorganisms.

Organism	RD	RDBNC	BNT	Antibiotic
(Gram positive) S.aureus ATTC29213	0.12 ± 0.03	0.25 ± 0.30	0.35 ± 0.11	1
(Gram Negative) *E.coli* ATCC 25922	0.25 ± 0.45	0.12 ± 0.22	0.17 ± 0.36	2
(Fungi) C.albicans ATCC 10231	0.06 ± 0.01	0.62 ± 0.67	0.18 ± 0.87	2

Values expressed are means ± SD of three parallel measurements.

The fact that RDBNC is more effective in Gram-negative bacteria may be related to the fact that Gram-positive bacteria have a hard polysaccharide layer in the cell wall of Gram-positive bacteria that is not present in Gram-negative bacteria, and therefore, it is difficult to penetrate the Gram-positive bacterial wall [64]. Although the mechanisms of action of the antimicrobial activity of nanomaterials are not fully known, it is supported by the literature that they may enter the microbe and by interacting with the sulfur and phosphorus group of the DNA/protein cause damage, or they might bind to the cytoplasmic membrane and destroy the bacterial cell due to the electrostatic affinity between the positively charged NPs and the negatively charged cell membrane of the microbe [65,66].

## 3 Conclusion

RDBNC bionanosorbent was synthesised by RD mushroom, bentonite clay with TTP to investigate the adsorption of MB, MG in batch mode process. FTIR, FESEM, and TGA characterized the chemical structure, morphology, and thermal properties of RDBNC. Also, the zeta-potential test was implemented to expose the surface ionicity of bionanosorbentand the crystallite size of RDBNC was calculated as 20 nm according to the Dybe Scherer Equation by XRD tecnique. The experiments explained that the number of dyes adsorbed primarily depended on the initial dye concentrations, bionanosorbent dose, and temperature. The adsorption capacity of MB and MG was found to be increased with increasing concentrations of dyes and bionanosorbent dose. However, with increasing temperature from 303 K to 323 K, both dye adsorption were decreased from 157.3 mg/g to 39.76 mg/g from 92.05 mg/g to 38.25 mg/g for MG, respectively that indicates the exothermic nature of adsorption. As a result of TGA analysis, it was reported that a more durable composite was obtained by adding clay. The results showed that the data was well-fitted with the Freundlich isotherm model that indicate the multilayer nature of the sorption process and the adsorption kinetics followed the pseudo second order model. It was observed that the adsorption mechanism involved was physical adsorption mainly due to hydrogen bonding, π–π interactions, and electrostatic interaction according to isotherm investigations. Additionally, the minus Zeta potential value (−11.3 mV) of the bionanosorbent supports electrostatic interaction with cationic dyes. Thermodynamic parameters suppose the adsorption tends to exhibit a higher degree of randomness at the solid-liquid interface, exothermic, and physical process for MB, and MG onto RDBNC. The RDBNC could be reused at least for the 4 cycles of adsorption/desorption without losing its adsorption properties for MB and MG dyes. The results proved that RDBNC showed outstanding adsorption towards both MB and MG dyes and strong antimicrobial activity even at low concentrations. In this context, it is clear that the newly synthesised bionanomaterial can be used for different applications. This simple, environmentally friendly, cost-effective, new innovative materyal RDBNC for bionanosorbent synthesis could provide excellent results in applications such as biotechnology, environment and biomedicine.

## Consent to participate

All authors consented to participate in this work.

## CRediT authorship contribution statement

**Ayfer Yildirim:** Writing – review & editing, Writing – original draft, Visualization, Validation, Supervision, Software, Resources, Project administration, Methodology, Investigation, Funding acquisition, Formal analysis, Data curation, Conceptualization. **Hilal Acay:** Writing – original draft, Visualization, Validation, Supervision, Software, Resources, Project administration, Methodology, Investigation, Funding acquisition, Formal analysis, Data curation, Conceptualization.

## Ethics approval

Not applicable.

## Consent for publication

All authors consent to publish this paper.

## Data availability

Data will be made available on request.

## Funding

This research was supported by the Mardin Artuklu University Scientific Research Projects Coordination Unit (Project number MAU.BAP.22.SBF.009).

## Declaration of competing interest

The authors declare that they have no known competing financial interests or personal relationships that could have appeared to influence the work reported in this paper.
